# Framingham Risk Score with Cardiovascular Events in Chronic Kidney Disease

**DOI:** 10.1371/journal.pone.0060008

**Published:** 2013-03-20

**Authors:** Szu-Chia Chen, Ho-Ming Su, Yi-Chun Tsai, Jiun-Chi Huang, Jer-Ming Chang, Shang-Jyh Hwang, Hung-Chun Chen

**Affiliations:** 1 Division of Nephrology, Department of Internal Medicine, Kaohsiung Medical University Hospital, Kaohsiung Medical University, Kaohsiung, Taiwan; 2 Division of Cardiology, Department of Internal Medicine, Kaohsiung Medical University Hospital, Kaohsiung Medical University, Kaohsiung, Taiwan; 3 Department of Internal Medicine, Kaohsiung Municipal Hsiao-Kang Hospital, Kaohsiung Medical University, Kaohsiung, Taiwan; 4 Faculty of Renal Care, College of Medicine, Kaohsiung Medical University, Kaohsiung, Taiwan; 5 Faculty of Medicine, College of Medicine, Kaohsiung Medical University, Kaohsiung, Taiwan; University of Sao Paulo, Brazil

## Abstract

The Framingham Risk Score (FRS) was developed to predict coronary heart disease in various populations, and it tended to under-estimate the risk in chronic kidney disease (CKD) patients. Our objectives were to determine whether FRS was associated with cardiovascular events, and to evaluate the role of new risk markers and echocardiographic parameters when they were added to a FRS model. This study enrolled 439 CKD patients. The FRS is used to identify individuals categorically as “low” (<10% of 10-year risk), “intermediate” (10–20% risk) or “high” risk (≧ 20% risk). A significant improvement in model prediction was based on the −2 log likelihood ratio statistic and c-statistic. “High” risk (*v.s.* “low” risk) predicts cardiovascular events either without (hazard ratios [HR] 2.090, 95% confidence interval [CI] 1.144 to 3.818) or with adjustment for clinical, biochemical and echocardiographic parameters (HR 1.924, 95% CI 1.008 to 3.673). Besides, the addition of albumin, hemoglobin, estimated glomerular filtration rate, proteinuria, left atrial diameter >4.7 cm, left ventricular hypertrophy or left ventricular ejection fraction<50% to the FRS model significantly improves the predictive values for cardiovascular events. In CKD patients, “high” risk categorized by FRS predicts cardiovascular events. Novel biomarkers and echocardiographic parameters provide additional predictive values for cardiovascular events. Future study is needed to assess whether risk assessment enhanced by using these biomarkers and echocardiographic parameters might contribute to more effective prediction and better care for patients.

## Introduction

Chronic kidney disease (CKD) is an increasingly noted worldwide public health problem associated with increased morbidity and mortality, and cardiovascular disease is a major cause in CKD patients [1], [2]. Such an excessive cardiovascular risk is in part attributed to the high prevalence of traditional risk factors among people with CKD, including hypertension, diabetes and dyslipidemia, but may also relate to presence of other non-traditional risk factors [3]–[7]. Identifying these risk factors and acquiring preventive and interventional strategies is an initial and essential step in managing patients with CKD.

The Framingham Risk Score (FRS) allows clinicians to estimate the individual patient risk of coronary heart disease by using traditional cardiac risk factors including age, gender, systolic blood pressure, treatment of hypertension, total cholesterol, high-density lipoprotein (HDL) cholesterol, and cigarette smoking [8]. In patients with CKD, non-traditional factors such as anemia, renal insufficiency, albuminuria, malnutrition and structural and functional abnormalities of heart are prevalent and may increase the risk of cardiovascular disease [3]–[7], [9]. It has been noticed that the traditional risk factors in CKD patients may differ from those in the general population [10]. Weiner et al. reported that FRS showed poor overall accuracy in predicting cardiac events in individuals with CKD [11]. Improvements in risk prediction other than FRS with new risk markers including metabolic syndrome, C-reactive protein, coronary artery calcium score, carotid intima-media thickness, ankle brachial index and pulse wave velocity have been reported in various populations [12]–[16]. Our recent study shows that echocardiographic parameters including left atrial size, left ventricular hypertrophy and left ventricular systolic function, help predict adverse cardiovascular outcomes in patients with CKD [17]. However, whether new risk markers can improve cardiovascular disease risk prediction besides FRS remains unclear in CKD. Our previous study showed that the prevalence of cardiovascular disease was up to 15.1% in moderate to advanced CKD [18]. Hence, the objectives of the present study are to determine (1) whether FRS is associated with cardiovascular events, and (2) whether the new biomarkers and echocardiographic parameters can be more predictive when they are added to the FRS model in CKD patients.

## Subjects and Methods

### Study Patients and Design

The study was conducted in a regional hospital in southern Taiwan. We consecutively recruited 518 pre-dialysis patients with stages 3 to 5 of CKD according to the National Kidney Foundation-Kidney Disease Outcomes Quality Initiative (K/DOQI) guidelines [19] from January 2007 to May 2010. We classified our patients with evidence of kidney damage lasting for more than 3 months into CKD stage 3, 4, and 5, based on estimated glomerular filtration rate (eGFR) level (mL/min/1.73 m^2^) of 30 to 59, 15 to 29, and <15 respectively. The study patients received regular follow-up at our Outpatient Department of Internal Medicine. They were selected to take part in this study if they agreed to receive echocardiographic examination. Three patients refused echocardiography examinations due to personal reasons. Five patients with significant mitral regurgitation and five others with inadequate image visualization were also excluded. Significant mitral regurgitation was defined as the regurgitant jet area more than 20% of the left atrial area [20]. To calculate FRS, we also exclude patients without HDL-cholesterol data (n = 5), and those with age older than 80 years (n = 61). Finally, a total of 439 patients were included.

### Ethics Statement

The study protocol was approved by the Institutional Review Board of Kaohsiung Medical University Hospital (KMUH-IRB-20110158). Informed consents were obtained in written forms, and all clinical investigations were conducted according to the principles expressed in the Declaration of Helsinki. The patients gave consent for the publication of the clinical details.

### Evaluation of Cardiac Structure and Function

The echocardiographic examinations were performed by the same cardiologist with a VIVID 7 (General Electric Medical Systems, Horten, Norway), with the participants resting in the left decubitus position. The examiner was blind to the biochemical data. Two-dimensional and two-dimensionally guided M-mode images were recorded from the standardized views. The echocardiographic measurements included left atrial (LA) diameter, left ventricular internal diameter in diastole (LVIDd), left ventricular posterior wall thickness in diastole (LVPWTd), interventricular septal wall thickness in diastole (IVSTd), peak early transmitral filling wave velocity (E) and peak late transmitral filling wave velocity (A). Left ventricular systolic function was assessed by left ventricular ejection fraction (LVEF). Left ventricular mass was calculated using the Devereux-modified method, i.e. left ventricular mass = 1.04 × [(IVSTd+LVIDd+LVPWTd)^3^– LVIDd^3^] –13.6g [21]. Left ventricular mass index (LVMI) was calculated by dividing left ventricular mass by body surface area. Left ventricular hypertrophy (LVH) was defined as suggested by the 2007 European Society of Hypertension/European Society of Cardiology Guidelines [22].

### FRS Score Calculation and Risk Category

The FRS was calculated based on a model comprised of age, gender, total cholesterol, HDL-cholesterol, systolic blood pressure, treatment of hypertension and cigarette smoking [8]. FRS identified individuals categorically as “low” (<10% 10-year risk), “intermediate” (10–20% risk) or “high” risk (≧ 20% risk).

### Collection of Demographic, Medical, and Laboratory Data

Demographic and medical data including age, gender, smoking history (current *versus* former), and co-morbid conditions were obtained from medical records or interviews with patients. Study subjects were defined as having diabetes mellitus (DM) if the claimed records had ICD-9 code 250.00 to 250.90, or the fasting blood glucose level was greater than 126 mg/dL, or hypoglycemic agents were used to control blood glucose levels. A similar definition was applied to hypertension with an ICD-9 code of 401.9, diagnosed by a physician, a systolic BP≥140 mmHg or diastolic BP≥90 mmHg, or using antihypertensive medications irrespective of BP. Cerebrovascular disease was defined as a history of cerebrovascular accident including cerebral bleeding and infarction. Coronary artery disease was defined as a history of angina, ischemic change on electrocardiogram, history of myocardial infarction, or having undergone coronary bypass surgery or angioplasty. The body mass index was calculated as the ratio of weight in kilograms divided by square of height in meters. Laboratory data were measured from fasting blood samples using an autoanalyzer (Roche Diagnostics GmbH, D-68298 Mannheim COBAS Integra 400). Serum creatinine was measured by the compensated Jaffé method (kinetic alkaline picrate) in a Roche/Integra 400 Analyzer (Roche Diagnostics, Mannheim, Germany) using a calibrator traceable to isotope-dilution mass spectrometry [23]. The value of eGFR was calculated using the 4-variable equation in the Modification of Diet in Renal Disease (MDRD) study [24]. Proteinuria was examined by dipsticks (Hema-Combistix, Bayer Diagnostics). A test result of 1+ or more was defined as positive. Blood and urine samples were obtained within 1 month of enrollment.

### Definition of Cardiovascular Events

Cardiovascular events were defined as cardiovascular death, hospitalization for unstable angina, nonfatal myocardial infarction, sustained ventricular arrhythmia, hospitalization for congestive heart failure, transient ischemia attack, and stroke. Cardiovascular events were ascertained and adjudicated by two cardiologists with disagreement resolved by adjudication from a third cardiologist from the hospital course and medical record. The study subjects were followed either until the first episode of cardiovascular events occurred or until February 2011.

### Statistical Analysis

Statistical analysis was performed using SPSS 15.0 for Windows (SPSS Inc. Chicago, USA). Data were expressed as percentages, mean ± standard deviation, or median (25^th^–75^th^ percentile) for triglyceride. The study patients were stratified into 3 groups according to FRS categories. Multiple comparisons among the study groups were performed by one-way analysis of variance (ANOVA) followed by post hoc test adjusted with a Bonferroni correction. Time to cardiovascular events and covariates of risk factors were modeled using the Cox proportional hazards model. A significant improvement in the Cox proportional hazards model prediction was based on the -2 log likelihood ratio statistic, which followed a difference in likelihood and the *P* value was based on the incremental value compared with the FRS model. A difference was considered significant if the *P* value was less than 0.05. To determine whether adding the new variable to FRS improved risk prediction, we used the c-statistic. The risk score was then used to calculate the predictive validity of each model using the receiver-operator characteristics (ROC) curve analysis [25].

## Results

The mean age of the 439 patients was 64.0±11.1 years. There were 143, 116 and 180 patients in the “low”, “intermediate” and “high” risk categories respectively. The comparison of baseline characteristics among patients with different FRS categories is shown in Table 1. Compared with patients with “low” risk, patients with “intermediate” risk were found to be older, more likely to be male, had higher prevalence of being a current smoker, and had lower LVEF. In addition, compared with patients with “low” risk, patients with “high” risk were older, more likely to be male, and had higher mean arterial pressure, higher pulse pressure, lower albumin, higher fasting glucose, lower HDL-cholesterol, higher low-density lipoprotein (LDL) cholesterol, higher LA diameter, higher LVMI, lower LVEF and lower E/A.

**Table 1:** Comparison of baseline characteristics among patients with different FRS category.

Characteristics	low risk (n = 143)	intermediate risk (n = 116)	high risk (n = 180)
Age (year)	56.4±10.7	63.9±9.3*	70.0±8.7* ^†^
Male gender (%)	24.5	75.0*	86.1*
Current smoker (%)	11.2	28.4*	52.8* ^†^
Diabetes mellitus (%)	49.7	52.6	66.7^†^
Hypertension (%)	77.6	80.2	87.8
Coronary artery disease (%)	10.5	11.2	13.3
Mean arterial pressure (mmHg)	97.0±12.5	98.0±14.0	103.8±13.9* ^†^
Pulse pressure (mmHg)	55.6±15.9	60.0±16.1	66.4±17.6* ^†^
Body mass index (kg/m^2^)	25.4±4.6	25.0±3.7	25.6±3.8
Laboratory parameters			
Albumin (g/dL)	4.12±0.30	4.00±0.47	3.97±0.40*
Fasting glucose (mg/dL)	122.2±54.1	116.5±42.5	137.0±71.6^†^
Triglyceride (mg/dL)	133 (93–188)	132.5 (90.5–193.5)	156 (101.5–224.75)
Total cholesterol (mg/dL)	193.2±48.9	196.5±48.1	196.8±47.0
HDL-cholesterol (mg/dL)	50.6±16.0	49.8±15.3	38.9±7.1* ^†^
LDL-cholesterol (mg/dL)	102.6±35.4	107.4±36.9	114.6±37.5*
Hemoglobin (g/dL)	11.3±2.2	11.6±2.2	12.0±2.6
eGFR (mL/min/1.73 m^2^)	24.8±14.9	27.3±14.4	27.1±14.1
Uric acid (mg/dL)	8.0±2.3	8.0±2.1	8.5±2.1
Proteinuria (%)	66.4	67.5	66.7
Echocardiographic data			
LA diameter (cm)	3.7±0.6	3.7±0.6	3.9±0.6* ^†^
LA diameter >4.7 cm (%)	4.2	2.6	10.6^†^
LVMI (g/m^2^)	129.2±42.2	137.2±48.3	154.4±47.8* ^†^
LVH (%)	60.8	61.2	72.8
LVEF (%)	70.7±9.0	66.2±12.2*	67.7±11.8*
LVEF<50% (%)	2.8	8.6	7.8
E/A	0.93±0.31	0.86±0.45	0.82±0.36*

Abbreviations: HDL, high-density lipoprotein; LDL, low-density lipoprotein; eGFR, estimated glomerular filtration rate; LA, left atrial; LV, left ventricular; LVMI, left ventricular mass index; LVH, left ventricular hypertrophy; LVEF, left ventricular ejection fraction; E, peak early transmitral filling wave velocity; A, peak late transmitral filling wave velocity.

The FRS is used to identify individuals categorically as “low” (<10% of 10-year risk), “intermediate” (10–20% risk), or “high” risk (≥20% risk).

* *P*<0.05 compared with low risk; ^†^ *P*<0.05 compared with intermediate risk.

### Relation of FRS Category to Cardiovascular Events

The mean follow-up period was 26.5±12.3 months (range 6–50 months). Seventy cardiovascular events (15.9%) were recorded during the follow-up period, including cardiovascular death (n = 17), hospitalization for unstable angina and nonfatal myocardial infarction (n = 16), sustained ventricular arrhythmia (n = 6), hospitalization for congestive heart failure (n = 16), and transient ischemia attack and stroke (n = 15). Table 2 shows the hazard ratios (HR) of the FRS category for cardiovascular events with and without adjustment for clinical, biochemical and echocardiographic parameters. Patients with “high” risk were significantly associated with cardiovascular events (HR 2.090, 95% confidence interval [CI] 1.144 to 3.818, *P* = 0.017 *v.s.* “low” risk), whereas patients with “intermediate” risk (*v.s.* “low” risk), did not achieve significance (*P* = 0.135). In addition, the univariate regression analysis showed that the presence of DM, coronary artery disease, low albumin, low hemoglobin, low eGFR, high uric acid, proteinuria, left atrial diameter >4.7 cm, left ventricular hypertrophy, and left ventricular ejection fraction <50% were all significantly associated with an increase in cardiovascular events. The relation of “high” risk patients to cardiovascular events still remained significant after further adjustment for diabetes mellitus, coronary artery disease, albumin, hemoglobin, eGFR, uric acid, proteinuria, left atrial diameter >4.7 cm, left ventricular hypertrophy and left ventricular ejection fraction <50% (HR 1.924, 95% CI 1.008 to 3.673, *P* = 0.047 *v.s.* “low” risk).

**Table 2:** Relation of FRS category to cardiovascular events using Cox proportional hazards model.

Parameters	Unadjusted		Multivariate adjusted	
	HR (95% CI)	*P*	HR (95% CI)	*P*
FRS risk category				
low risk	1		1	
intermediate risk	1.677 (0.852–3.300)	0.135	1.783 (0.860–3.697)	0.120
high risk	2.090 (1.144–3.818)	0.017	1.924 (1.008–3.673)	0.047
Diabetes mellitus	2.665 (1.522–4.665)	0.001	1.580 (0.875–2.852)	0.129
Coronary artery disease	3.295 (1.944–5.586)	<0.001	3.639 (2.094–6.326)	<0.001
Albumin (g/dL)	0.255 (0.162–0.401)	<0.001	0.377 (0.205–0.696)	0.002
Hemoglobin (g/dL)	0.771 (0.694–0.856)	<0.001	0.801 (0.683–0.939)	0.006
eGFR (mL/min/1.73 m^2^)	0.963 (0.945–0.981)	<0.001	1.000 (0.968–1.033)	0.993
Uric acid (mg/dL)	1.124 (1.016–1.243)	0.023	1.063 (0.953–1.186)	0.275
Proteinuria	2.293 (1.254–4.194)	0.007	0.895 (0.398–2.016)	0.790
LA diameter>4.7 cm	4.040 (2.164–7.543)	<0.001	1.607 (0.805–3.210)	0.179
LVH	3.717 (1.845–7.487)	<0.001	2.028 (0.976–4,215)	0.058
LVEF <50%	3.451 (1.808–6.586)	<0.001	1.640 (0.802–3.353)	0.176

Values express as hazard ratios (HR) and 95% confidence interval (CI).

Abbreviations are the same as in Table 1.

**Figure 1:**
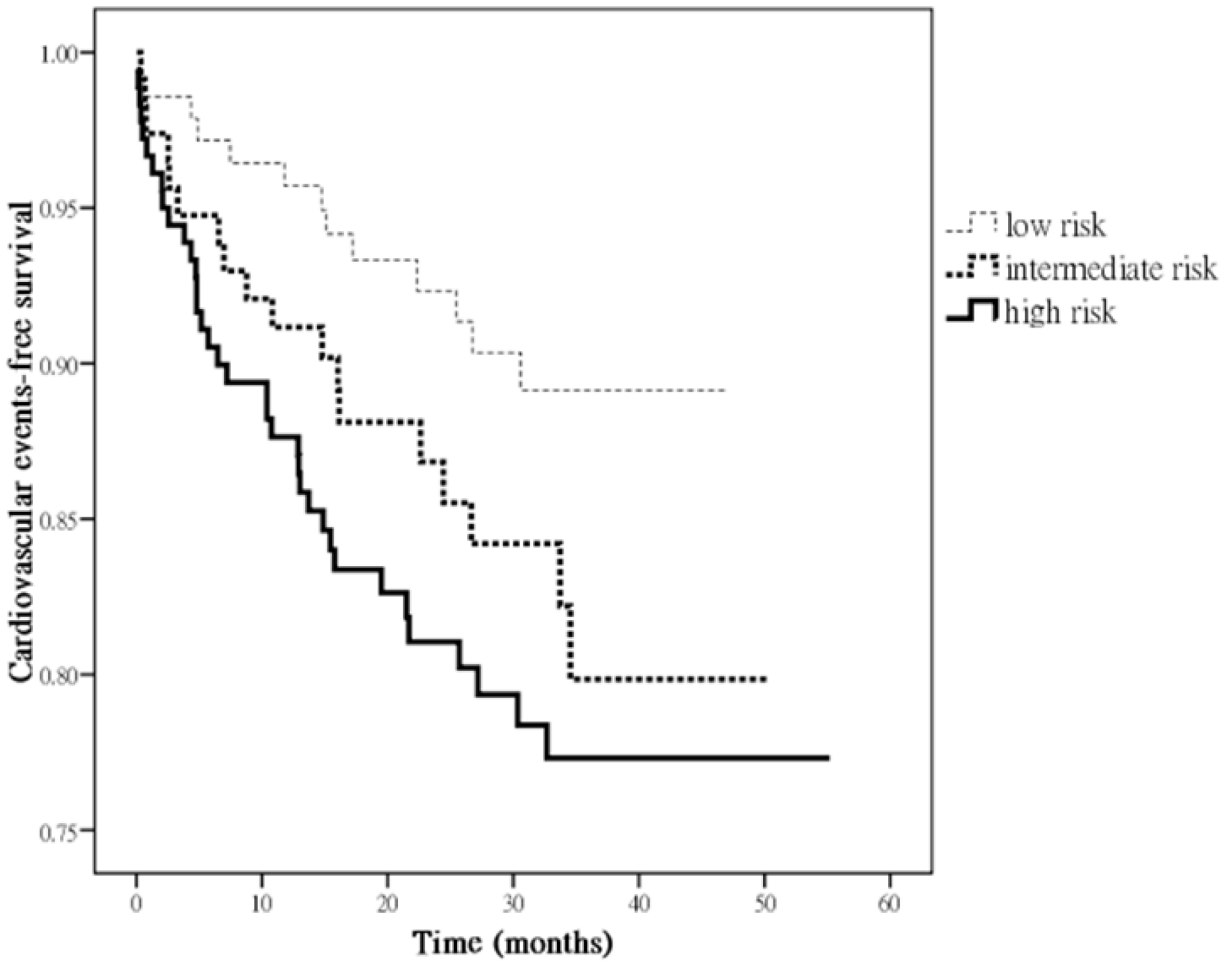
Kaplan-Meier analyses of cardiovascular event-free survival in all patients subdivided according to different FRS category. Patients with “high” risk, but not “intermediate” risk (*vs*. “low” risk) were significantly associated with increased cardiovascular event (*P* = 0.039).

Figure 1 illustrates the Kaplan-Meier curves for cardiovascular event-free survival in all patients subdivided by different FRS category. Patients with “high” risk, but not “intermediate” risk (*v.s*. “low” risk) were significantly associated with increased cardiovascular event (*P* = 0.039).

### Incremental Values of Biomarkers and Echocardiographic Parameters for Cardiovascular Events

The incremental values of biomarkers and echocardiographic parameters, when they were added to the FRS model in prediction of cardiovascular events, are shown in Table 3. The addition of albumin (*P*<0.001), hemoglobin (*P*<0.001), eGFR (*P*<0.001), and proteinuria (*P*<0.001) to the FRS model significantly improved the predictive values of cardiovascular events. Similarly, the addition of LA diameter >4.7 cm (*P*<0.001), LVH (*P*<0.001) or LVEF <50% (*P* = 0.003) to the FRS model also significantly improved the predictive values of cardiovascular events.

**Table 3:** Incremental values of biomarkers and echocardiographic parameters for cardiovascular events when added to the FRS model using Cox proportional hazards model.

Parameters	Cardiovascular events	
	difference inlikelihood	*P*
FRS + albumin	32.651	<0.001
FRS + hemoglobin	28.679	<0.001
FRS + eGFR	20.401	<0.001
FRS + proteinuria	21.053	<0.001
FRS + LA diameter >4.7 cm	12.240	<0.001
FRS + LVH	17.030	<0.001
FRS + LVEF <50%	9.082	0.003

*P* value was based on the incremental value compared with the FRS model.

Abbreviations are the same as in Table 1.

Table 4 summarizes the changes in c-statistic and ROC curves analysis with calculated area under the curve (AUCs) when each new risk marker was added to the base model. The maximum change in c-statistic was observed for albumin (increase, 0.204), followed by hemoglobin, eGFR, LA diameter >4.7 cm, LVH, proteinuria, and LVEF <50%.

**Table 4:** Prognostic value of biomarkers and echocardiographic parameters for cardiovascular events using c-statistic and ROC curve analysis with calculated AUCs.

Parameters	Cardiovascular events	
	AUCs	Change in c-statistic
FRS	0.589	
FRS + albumin	0.793	0.204
FRS + hemoglobin	0.768	0.179
FRS + eGFR	0.756	0.167
FRS + proteinuria	0.678	0.089
FRS + LA diameter >4.7 cm	0.725	0.136
FRS + LVH	0.700	0.111
FRS + LVEF <50%	0.648	0.059

Abbreviations: ROC, receiver operating characteristic; AUC, area under the curve; eGFR, estimated glomerular filtration rate; LA, left atrial; LVH, left ventricular hypertrophy; LVEF, left ventricular ejection fraction.

## Discussion

In the present study, we evaluated the association between FRS categories and cardiovascular events and whether novel biomarkers and echocardiographic parameters added to the FRS model could improve the predictive value of FRS for cardiovascular events in CKD patients over a follow-up period of 2.2 years. We found that “high” risk patients (*v.s.* “low” risk) were significantly associated with more cardiovascular events. Besides, the addition of albumin, hemoglobin, eGFR, proteinuria, LA diameter >4.7 cm, LVH or LVEF <50% to the FRS model could significantly improve the predictive values for cardiovascular events in CKD patients.

The FRS is the most commonly used coronary heart disease risk prediction instrument in clinical settings by counting traditional cardiac risk factors including age, sex, systolic blood pressure, treatment of hypertension, total cholesterol, HDL-cholesterol and cigarette smoking [8]. FRS is used to quantify 10-year risk. Individuals with “high” risk (>20% of 10-year risk of coronary heart disease) are considered “coronary heart disease equivalent” and are candidates for intensive medical risk reduction and those at “intermediate” risk (>6 and ≤20% of 10-year risk) are candidates for less intensive risk factor reduction [8]. In our study, we also found that “high” risk categorized by FRS was significantly associated with cardiovascular events in CKD patients. Therefore, CKD patients, with “high” risk may need intensive medical risk reduction, as expected.

It has been discussed that traditional methods of risk adjustment might have limitations in the CKD patients [17], [26], [27]. The FRS was created using data from a population-based cohort in which the majority did not have CKD. Thus, the application of FRS in patients with CKD deserves careful evaluation and revision. In patients with renal insufficiency, other nontraditional factors were prevalent and might increase the risk of cardiovascular disease [17], [26], [27]. The excessive cardiac risk associated with CKD has been evaluated by adding eGFR, cystatin C, serum creatinine and albuminuria to the Framingham equations [11], [28], [29]. Increased levels of urine albumin excretion and decreased eGFR were shown to anticipate increased cardiac risk in CKD patients [1], [30]. In the present study, albumin, uric acid and proteinuria provided additional predictive values for cardiovascular events when added to the FRS model, which supported previous studies [30]. However, addition of several new variables to a risk prediction tool might take part of the convenience away, although it could improve the predictive value in CKD patients.

Structural and functional abnormalities of the heart in CKD patients were frequently noticed because of persistent pressure and volume overload [31], [32]. Echocardiographic measures of left ventricular function and structure as well as LA size have been reported to predict adverse cardiovascular outcomes in a variety of populations [17], [33]–[36]. Our recent study demonstrated LA diameter >4.7 cm, LVH and LVEF <50% could predict adverse cardiovascular events in CKD. In the present study, we found the addition of LA diameter >4.7 cm (*P*<0.001), LVH (*P*<0.001) or LVEF <50% (*P* = 0.003) to the FRS model could significantly improve the predictive values for cardiovascular events other than FRS alone. Screening CKD patients by means of FRS and echocardiographic parameters might help identify a high risk group with poorer cardiovascular outcomes. Patients with significant mitral regurgitation were excluded because mitral regurgitation may influence the measurements of LA diameter, and our results could not be applied in these patients.

In conclusion, our results demonstrated that “high” risk categorized by FRS could predict cardiovascular events in CKD patients. Furthermore, albumin, hemoglobin eGFR, proteinuria, LA diameter >4.7 cm, LVH and LVEF <50% could improve the predictive values of cardiovascular events when added to the FRS model. Further investigation is needed to assess whether risk prediction using these biomarkers and echocardiographic parameters could lead to more intensive care in the susceptible groups of patients and improve their clinical outcomes.

## References

[1] Go AS, Chertow GM, Fan D, McCulloch CE, Hsu CY (2004) Chronic kidney disease and the risks of death, cardiovascular events, and hospitalization. N Engl J Med 351: 1296–1305. 15385656 10.1056/NEJMoa041031

[2] Tonelli M, Wiebe N, Culleton B, House A, Rabbat C, et al (2006) Chronic kidney disease and mortality risk: A systematic review. J Am Soc Nephrol 17: 2034–2047. 16738019 10.1681/ASN.2005101085

[3] Olivero JJ, Nguyen P (2009) Chronic kidney disease: A marker of cardiovascular disease. Methodist Debakey Cardiovasc J 5: 24–29. 20073162 10.14797/mdcj-5-2-24

[4] Rodriguez-Iturbe B, Correa-Rotter R (2010) Cardiovascular risk factors and prevention of cardiovascular disease in patients with chronic renal disease. Expert Opin Pharmacother 11: 2687–2698. 20426701 10.1517/14656561003796570

[5] Zoccali C, Benedetto FA, Tripepi G, Mallamaci F, Rapisarda F, et al (2006) Left ventricular systolic function monitoring in asymptomatic dialysis patients: A prospective cohort study. J Am Soc Nephrol 17: 1460–1465. 16597683 10.1681/ASN.2005111240

[6] Silberberg JS, Barre PE, Prichard SS, Sniderman AD (1989) Impact of left ventricular hypertrophy on survival in end-stage renal disease. Kidney Int 36: 286–290. 2528654 10.1038/ki.1989.192

[7] Cintron G, Johnson G, Francis G, Cobb F, Cohn JN (1993) Prognostic significance of serial changes in left ventricular ejection fraction in patients with congestive heart failure. The v-heft va cooperative studies group. Circulation 87: VI17–23. 8500235

[8] Third report of the national cholesterol education program (ncep) expert panel on detection, evaluation, and treatment of high blood cholesterol in adults (adult treatment panel iii) final report. Circulation 106: 3143–3421. 12485966

[9] Sarnak MJ, Levey AS (2003) Schoolwerth AC, Coresh J, Culleton B, et al (2003) Kidney disease as a risk factor for development of cardiovascular disease: A statement from the american heart association councils on kidney in cardiovascular disease, high blood pressure research, clinical cardiology, and epidemiology and prevention. Hypertension 42: 1050–1065. 14604997 10.1161/01.HYP.0000102971.85504.7c

[10] Kalantar-Zadeh K, Block G, Humphreys MH, Kopple JD (2003) Reverse epidemiology of cardiovascular risk factors in maintenance dialysis patients. Kidney Int 63: 793–808. 12631061 10.1046/j.1523-1755.2003.00803.x

[11] Weiner DE, Tighiouart H, Elsayed EF, Griffith JL, Salem DN, et al (2007) The framingham predictive instrument in chronic kidney disease. J Am Coll Cardiol 50: 217–224. 17631213 10.1016/j.jacc.2007.03.037

[12] Wannamethee SG, Shaper AG, Lennon L, Morris RW (2005) Metabolic syndrome vs framingham risk score for prediction of coronary heart disease, stroke, and type 2 diabetes mellitus. Arch Intern Med 165: 2644–2650. 16344423 10.1001/archinte.165.22.2644

[13] Mattace-Raso FU, van der Cammen TJ, Hofman A, van Popele NM, Bos ML, et al (2006) Arterial stiffness and risk of coronary heart disease and stroke: The rotterdam study. Circulation 113: 657–663. 16461838 10.1161/CIRCULATIONAHA.105.555235

[14] Mitchell GF (2004) Increased aortic stiffness: An unfavorable cardiorenal connection. Hypertension 43: 151–153. 14718350 10.1161/01.HYP.0000114581.77705.29

[15] Fowkes FG, Murray GD, Butcher I, Heald CL, Lee RJ, et al (2008) Ankle brachial index combined with framingham risk score to predict cardiovascular events and mortality: A meta-analysis. JAMA 300: 197–208. 18612117 10.1001/jama.300.2.197 PMC2932628

[16] Kavousi M, Elias-Smale S, Rutten JH, Leening MJ, Vliegenthart R, et al (2012) Evaluation of newer risk markers for coronary heart disease risk classification: A cohort study. Ann Intern Med 156: 438–444. 22431676 10.7326/0003-4819-156-6-201203200-00006

[17] Chen SC, Chang JM, Liu WC, Huang JC, Tsai JC, et al (2012) Echocardiographic parameters are independently associated with increased cardiovascular events in patients with chronic kidney disease. Nephrol Dial Transplant 27: 1064–1070. 21813825 10.1093/ndt/gfr407

[18] Liu WC, Hung CC, Chen SC, Yeh SM, Lin MY, et al (2012) Association of hyperuricemia with renal outcomes, cardiovascular disease, and mortality. Clin J Am Soc Nephrol 7: 541–548. 22300737 10.2215/CJN.09420911

[19] Levey AS, Coresh J, Bolton K, Culleton B, Harvey KS, et al (2002) K/DOQI clinical practice guidelines for chronic kidney disease: evaluation, classification, and stratification. Am J Kidney Dis 39: S1–266. 11904577

[20] Helmcke F, Nanda NC, Hsiung MC, Soto B, Adey CK, et al (1987) Color Doppler assessment of mitral regurgitation with orthogonal planes. Circulation 75: 175–183. 3791603 10.1161/01.cir.75.1.175

[21] Devereux RB, Alonso DR, Lutas EM, Gottlieb GJ, Campo E, et al (1986) Echocardiographic assessment of left ventricular hypertrophy: Comparison to necropsy findings. Am J Cardiol 57: 450–458. 2936235 10.1016/0002-9149(86)90771-x

[22] Mancia G, De Backer G, Dominiczak A, Cifkova R, Fagard R, et al (2007) 2007 guidelines for the management of arterial hypertension: The task force for the management of arterial hypertension of the european society of hypertension (esh) and of the european society of cardiology (esc). J Hypertens 25: 1105–1187. 17563527 10.1097/HJH.0b013e3281fc975a

[23] Vickery S, Stevens PE, Dalton RN, van Lente F, Lamb EJ (2006) Does the id-ms traceable mdrd equation work and is it suitable for use with compensated jaffe and enzymatic creatinine assays? Nephrol Dial Transplant 21: 2439–2445. 16720592 10.1093/ndt/gfl249

[24] Levey AS, Bosch JP, Lewis JB, Greene T, Rogers N, et al (1999) A more accurate method to estimate glomerular filtration rate from serum creatinine: A new prediction equation. Modification of diet in renal disease study group. Ann Intern Med 130: 461–470. 10075613 10.7326/0003-4819-130-6-199903160-00002

[25] Uno H, Cai T, Pencina MJ, D’Agostino RB, Wei LJ (2011) On the C-statistics for evaluating overall adequacy of risk prediction procedures with censored survival data. Statistics in Medicine 30: 1105–1117. 21484848 10.1002/sim.4154 PMC3079915

[26] Chen SC, Chang JM, Hwang SJ, Tsai JC, Liu WC, et al (2010) Ankle brachial index as a predictor for mortality in patients with chronic kidney disease and undergoing haemodialysis. Nephrology (Carlton) 15: 294–299. 20470297 10.1111/j.1440-1797.2010.01187.x

[27] Knight EL, Rimm EB, Pai JK, Rexrode KM, Cannuscio CC, et al (2004) Kidney dysfunction, inflammation, and coronary events: A prospective study. J Am Soc Nephrol 15: 1897–1903. 15213279 10.1097/01.asn.0000128966.55133.69

[28] Hallan S, Astor B, Romundstad S, Aasarod K, Kvenild K, et al (2007) Association of kidney function and albuminuria with cardiovascular mortality in older vs younger individuals: The hunt ii study. Arch Intern Med 167: 2490–2496. 18071172 10.1001/archinte.167.22.2490

[29] Zethelius B, Berglund L, Sundstrom J, Ingelsson E, Basu S, et al (2008) Use of multiple biomarkers to improve the prediction of death from cardiovascular causes. N Engl J Med 358: 2107–2116. 18480203 10.1056/NEJMoa0707064

[30] Levey AS, de Jong PE, Coresh J, El Nahas M, Astor BC, et al (2011) The definition, classification, and prognosis of chronic kidney disease: A kdigo controversies conference report. Kidney Int 80: 17–28. 21150873 10.1038/ki.2010.483

[31] Paoletti E, Bellino D, Cassottana P, Rolla D, Cannella G (2005) Left ventricular hypertrophy in nondiabetic predialysis ckd. Am J Kidney Dis 46: 320–327. 16112052 10.1053/j.ajkd.2005.04.031

[32] Stewart GA, Gansevoort RT, Mark PB, Rooney E, McDonagh TA, et al.. (2005) Electrocardiographic abnormalities and uremic cardiomyopathy. Kidney Int 67:2 17–226. 10.1111/j.1523-1755.2005.00072.x 15610245

[33] Eckardt KU, Scherhag A, Macdougall IC, Tsakiris D, Clyne N, et al (2009) Left ventricular geometry predicts cardiovascular outcomes associated with anemia correction in ckd. J Am Soc Nephrol 20: 2651–2660. 19850955 10.1681/ASN.2009060631 PMC2794228

[34] Kim SJ, Han SH, Park JT, Kim JK, Oh HJ, et al (2011) Left atrial volume is an independent predictor of mortality in capd patients. Nephrol Dial Transplant 26: 3732–3739. 21430181 10.1093/ndt/gfr118

[35] Ristow B, Ali S, Whooley MA, Schiller NB (2008) Usefulness of left atrial volume index to predict heart failure hospitalization and mortality in ambulatory patients with coronary heart disease and comparison to left ventricular ejection fraction (from the heart and soul study). Am J Cardiol 102: 70–76. 18572038 10.1016/j.amjcard.2008.02.099 PMC2789558

[36] Silaruks S, Sirivongs D, Chunlertrith D (2000) Left ventricular hypertrophy and clinical outcome in capd patients. Perit Dial Int 20: 461–466. 11007379

